# Pediatric Health Mobility: Is it Only an Italian Problem?

**Published:** 2012-10-11

**Authors:** Giulia Paolella

**Affiliations:** 1 Medical school, University of Salerno, Salerno, Italy

**Keywords:** children, mobility, pediatric diseases

## Abstract

Intra-regional, extra-regional and international health mobility are important phenomena for regional and national healthcare planning. Pediatric data on this topic are scarce. We therefore conducted a systematic literature search on the PubMed database. Because of the insufficiency of published data we also resorted to conference proceedings and publications retrieved by Google Scholar and Google search engines. Thirty-one articles were identified. Main components of patients mobility were looking for better quality and timely treatment, advanced technology, expertise, and major organization. Our analysis highlights that pediatric mobility causes relevant medical, sociological and financial consequences.

## I. INTRODUCTION

Health mobility far from own residency is a well known phenomenon concerning both adults and children of any medical specialty. It involves the transfer of patients and resources between the nation and/or the region in which the patient lives and the one in which the target hospital is located. Italian healthcare system is a regionally based National Health Service that provides universal coverage generally free of charge at the point of service [**1**]. Different components of interregional mobility can almost always be traced back to the following components: programmed (due to the planned admission in extra-regional high specialized hospital), random component (when patient are abroad for other reason), border mobility (when occurs close to the regional border), suffered component (due to a lack of specialized care).

International mobility, a more numerically limited phenomenon, is caused to programmed cross-border care (e.g. travelling abroad for plastic surgery or dental care package); casual/occasional mobility (when patient is abroad for work, study, or vacation). In our Country there are also 3 specific situations of cross-border mobility: Vatican state (especially for the Pediatric Hospital Bambino Gesù of Rome), San Marino state and the municipality of Campione d’Italia in Lombardia region (but located in Swiss territory) **[2]**.

In Europe, the rights to international health mobility are likely to be changed by the new European Directive on cross-border mobility, which tends to favor patient mobility with predictable consequences for some relevant sectors (e.g. dental care) or for some countries (e.g. Eastern Europe) [**2–5**].

Determinants of extra-regional mobility are different and related to structural and professional deficiencies. Waiting lists and consequently long waiting periods, according to the survey of the Forum for biomedical research (year 2009), represent in Italy the main factors that encourage health mobility (over 72.8%). In fact, patients are willing to move to another region for an important health problem (39.6%), and this percentage increases ( 48.2%) for the Italian southern regions **[6]**.

Pediatric mobility has been scarcely studied, even if it is felt to be still a relevant problem in Campania region and in the other southern Italy regions. Although some previous pediatric studies [**7]** have been conducted to examine the main causes that underlie South-North Italian pediatric mobility, it seems that nowadays many families are still obliged to resort to extra-regional pediatric hospitals to receive an adequate medical treatment for their children, probably because the proposed corrective strategies were not always carried out.

The aim of this study is therefore to evaluate different aspects of pediatric health mobility and to review precedent analyses and approaches to this phenomenon.

## II. METHODOLOGY

The analysis of heath mobility was carried out by a systematic literature search on PubMed database. Most retrieved data regarded adult population. Although they were out of the primary scope of this work, were considered for better understanding of the phenomenon. Also conference proceedings and articles recovered by Google and Google Scholar search engines were therefore also considered. No language and publication restriction were imposed. The electronic literature search was performed using the following keywords: children, pediatric patients, mobility, migration, Italy regions, healthcare systems.

## III. RESULTS

We found thirty Italian (n=16), European (n=10), and North American (n=4) articles, the majority of them regarding international and interregional aspects of the general phenomenon mostly in adults(**Table 1**), with few data for pediatric population (**Table 2**).

**Figure 1.**
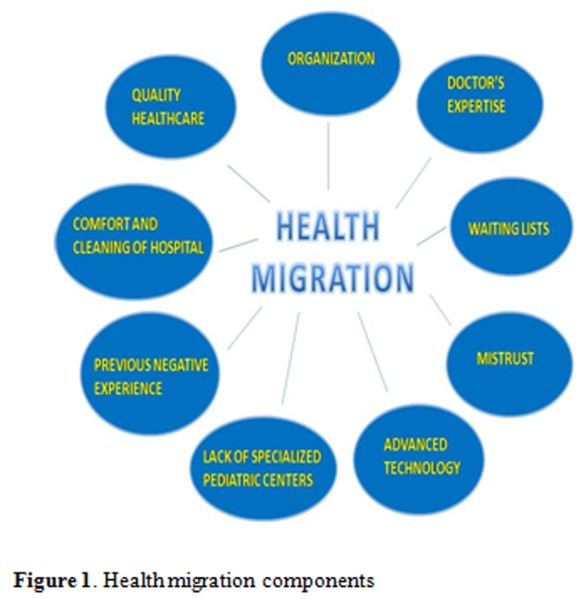
Health migration components

Summing up, several articles agree on most of health mobility components that causes the “journeys of hope” which are illustrated in **figure 1**.

Pediatric studies were available only on search-engines other than PubMed and regarded only Italy, where this phenomenon seems therefore to represent a peculiar problem.

Data for total (adults and children) Italian health mobility, regarding the year 2009, resulted in one billion and seventy five million Euros paid for extra-regional admissions from southern regions to hospitals located in northern areas [**23]**, confirming a previous Italian article which showed that patients mobility was mainly directed from the southern Italy regions to the North [**30**].

As shown in **Table 2**, pediatric information refer only to very little and dated evidence[**7;31–33**] and/or mainly available in the form of conference abstracts related to the general phenomenon [**34–37**], or specific pediatric subspecialties [**38].**

More recent data on pediatric mobility in Campania region showed that the main causes of extra-regional mobility were nervous system diseases (12.5%), followed by musculoskeletal and connective tissue disorders (12.2%), ear, nose and mouth diseases (8.2%), renal-urinary diseases (7.9%), myeloproliferative (7.6%) and mental disorders(7.4%) [**36**].

**Figure 2:**
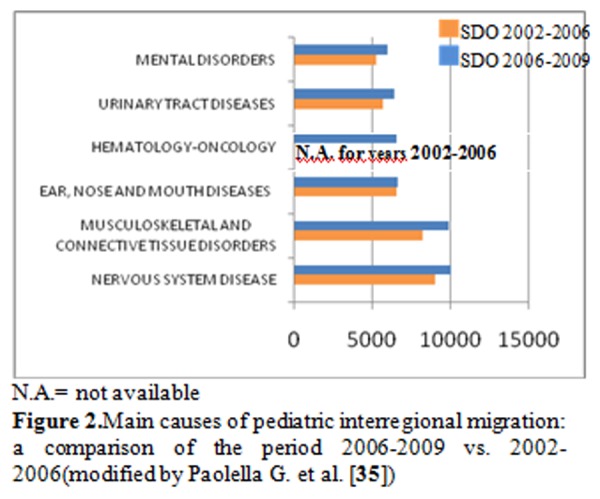
Main causes of pediatric interregional migration: a comparison of the period 2006–2009 vs. 2002–2006(modified by Paolella G. et al. [**35**])

Pediatric data compared with a previous study based on hospital discharge records (years 2002–2006) extracted from the regional archive of the Health Agency (ArSan) of Campania region [**34]** highlight that the major causes of pediatric mobility in 2002–2006 years were nervous system disease, upper respiratory tract disorders, renal-urinary diseases, hematology-oncology, musculoskeletal and connective tissue disorders. Unfortunately, nervous system diseases continue to represent the most important cause of extra-regional children mobility from Campania region (**Figure 2**).

Pediatric flows from all provinces of Campania region were mainly directed to the Pediatric Hospital Bambino Gesù of Rome (38.4%), and other hospitals of Lazio (14.3%), Tuscany (10.9%), Liguria (8.2%), Lombardy (7.1%) and Emilia Romagna (6.5%)regions. Border passivemobility other than for Lazio region had a low influence [Apulia(2.6%), Molise (2.5%), Basilicata region (2%)].

Greco and colleagues [**7**] examined pediatric mobility from the southern Italy regions to North Italy in 1982. In 50% of cases, extra-regional hospital admissions were spontaneous. The reasons for the initial mobility consisted in the lack of specialized centers in Southern Italy, and eventually previous negative experiences. About 30–40% of migrated sick children were affected by low-medium complexity disease, at the limit of the real need of hospitalization. Unfortunately, avoidable mobility continues to be a relevant component of the migratory flows **[36]**.

## IV. DISCUSSION AND CONCLUSION

Data shown above suggest that changing agreements between regions in order to discouragepatients mobility for low and medium complexity conditionsmay be necessary. Novel agreements between southern Italy regions with high percentage of extra-regional passive mobility and strongly attractive northern Italy regions need to be organized [**25**]. These programs of integrated services among regions (which are already in force in the northern Italy regions) would avoid duplication of health services and therefore optimize resources.

It should be noted that, nevertheless, health mobility is an unavoidable phenomenon for highly specialized healthcare. Since this phenomenon represents a significant cost to the Regional and National Health Systems, subtracting economical resources and inhibiting local healthcare growth, it will be necessary to recognize the different aspects of mobility to propose appropriate solution strategies. This is particularly critical in Italy, where the solution strategies proposed in the past were unsuccessful.

Patients attraction to South Italy regions’ hospitals could be achieved by acting on all of the different determinants that cause mobility: search for highly specialized centers, waiting lists, doctors’ expertise. As for as Pediatrics is concerned, establishing new specialized pediatric units/hospitals or centers of excellence might be a way out [**26]**. This is the recent case of the Mediterranean Pediatric Cardiology Centre (CCPM) in Sicily, a dislocated center of the Vatican City’s Pediatric Hospital Bambino Gesù of Rome: in this manner the Sicilian Region hopes to reach a reduction of extra-regional mobility for heart disease. Other accordances with Pediatric Hospital Bambino Gesùhave recently been stipulated with Calabria, Molise and Campania regions, but results are still to be evaluated. International (e.g. University of Pittsburgh) experimentation of Telemedicine for Radiology and Pathological anatomy at ISMETT of Palermo is also under evaluation [**49**].

It should be noted that further pediatric studies are needed to expand the database available for the Italian phenomenon of pediatric mobility. An accurate portrait of this phenomenon may be furnished by strict collaboration with regional health agencies. A survey of pediatric subspecialties and resources may allow to verify the possible role played by lack of information on existing resources, and to reduce at least the avoidable component of patients mobility.

## Figures and Tables

**Table 1:** North American and European articles/ conferences proceedings on health mobility

**Author, year, ref.**	**State**	**Topic of the study**
Geraedts, 2007 **[8]**	Germany	Quality hospital indicators
Cantarero, 2006 **[9]**	Spain	Analysis of mobility variables in Spanish regions
García-Lacalle, 2011, **[10]**	Spain	Patients satisfaction for healthcare
Brouwer, 2003 **[11]**	Netherlands	Waiting lists in Netherlands and cross-border mobility
Appleby, 2002 **[12]**	United Kingdom	Patient’s free choice of hospital and waiting lists
Propper, 2002 **[13]**	United Kingdom	Economical aspects in healthcare
Kanavos, 2000 **[14]**	E. U.	New directive of cross-border mobility in European Union.
Hermans, 2000 **[15]**	E.U.	Cross-border mobility in European Union and in particular for Germany, Belgium and Netherlands.
Ansell, 1998 **[16]**	Chicago, USA	Healthcare quality, and motivations of admissions in Cook County Hospital.
Luft, 1990 **[17]**	California, USA	Healthcare quality, and patient’s choice of hospital.
Tai, 2004 **[18]**	California, USA	Patient’s choice of hospital in rural areas
Tessier, 1985 **[19]**	Quebec, Canada	Healthcare resources distribution and avoidable mobility
Palm and Glinos, 2009 **[3]**	E.U.	Cross-border mobility in European Union
Brekke, 2011 **[20]**	E.U.	Healthcare quality, and Welfare
Lo Scalzo, 2009 [**1**]	Italy	Italian Health system review
Levaggi, 2004 **[21]**	Italy	Interregional patients migration
Porcu, 2007 **[22]**	Sardinia, Italy	A multi-way analysis on health mobility in Sardinia region
Proceeding Conference “Travel for Health. Healthmobility” **[2;6;23–29]**	Rome, Italy	General aspects and sociological variables of Italian Health mobility

**Table 2:** Italian pediatric studies and abstracts

**Author, year, ref.**	**Country**	**Topic of the study**
Greco, 1982–85, **[7;39–40]**	Italy	South-North migration in Italy
Grimaldi, 1983 **[31]**	Italy	Migration from southern Italy region
D’Andrea, 1992–93 [**33;41**]	Italy	Features of pediatric migration
Tamburlini, 1997 **[32]**	Italy	Pediatric mobility from Calabria region
La Gamba, 1999 **[42]**	Italy	Migration from southern Italy region
Marchetti, 2000 **[43]**	Italy	Quality healthcare for children with chronic diseases
de Campora, 2002 **[44–45]**	Italy	Health needs through SDO and anti-mobility analysis
Andria, 2007 [34]	Italy	Pediatric migration from Campania region
Pizzuti, 2008 **[46]**	Italy	Migration from Campania region
Vajro, 2011 **[35]**	Italy	Escape from hospitals
Paolella, 2011–12 **[36;47]**	Italy	Medical and socio-economic variables of health migration
Miniero, 2012 **[38]**	Italy	Pediatric migration for oncological diseases
Parisi, 2012 **[37;48]**	Italy	Pediatric migration from Calabria region
